# Development of JEV NS1 Specific Capture-ELISA Based on a Single Monoclonal Antibody

**DOI:** 10.3390/ani16162601

**Published:** 2026-08-20

**Authors:** Shu-Jian Zhang, Jian-Hui Zhang, Shi-Meng Liu, Yu-Ting Huang, Jin-Liang Wang, Zhi-Gao Bu, Rong-Hong Hua

**Affiliations:** 1 State Key Laboratory of Animal Disease Control and Prevention, Harbin Veterinary Research Institute, Chinese Academy of Agricultural Sciences, Harbin 150069, China; 2 Jiangsu Co-Innovation Centre for Prevention and Control of Important Animal Infectious Disease and Zoonoses, Yangzhou University, Yangzhou 225009, China

**Keywords:** Japanese encephalitis virus, NS1 protein, monoclonal antibody, antigen-capture ELISA

## Abstract

Japanese encephalitis virus (JEV) is a zoonotic pathogen transmitted primarily by Culex mosquitoes and causes severe neurological diseases in humans and animals. The main endemic areas are the Western Pacific and Southeast Asia, and its geographical distribution has expanded in recent years. The development of a diagnosis for orthoflavivirus infections is hampered by two main problems: the short duration of viremia, resulting in a narrow detection window, and severe cross-reactivity. NS1, a secreted nonstructural protein of orthoflavivirus, holds promise as a new target for overcoming these limitations. In this study, we established a highly specific and sensitive capture ELISA for the JEV NS1 protein. First, the JEV NS1 protein was successfully expressed in mammalian cells and purified using affinity chromatography. Seven mAbs recognizing JEV NS1 were generated, and the mAb 20B6 exhibited the strongest binding affinity. Based on 20B6, a capture ELISA was developed with an optimal coating concentration of 3 μg/mL and an optimal detection antibody working concentration of 0.432 μg/mL. No cross-reactivity was observed with other orthoflaviviruses (including WNV, KUNV, USUV, MVEV, SLEV, and ZIKV) or common porcine viruses. The method could effectively detect NS1 protein in cell culture medium, cell lysates, mouse tissues, and porcine serum samples from JEV-infected subjects. This study provides a solid foundation that may be further developed into an efficient and specific tool for epidemiological surveillance of Japanese encephalitis.

## Simple Summary

Similar to most orthoflaviviruses, the diagnosis of JEV infection is hindered by two major limitations: a narrow detection window due to transient viremia and extensive cross-reactivity with other orthoflaviviruses, such as West Nile virus (WNV) and dengue virus (DENV). To address these challenges, we developed a capture ELISA based on the stable dimeric form of the JEV NS1 protein with a single, highly specific monoclonal antibody (20B6). Our method exhibits no cross-reactivity with other orthoflaviviruses or common porcine viruses. Given the co-circulation of multiple orthoflaviviruses in regions such as China and Southeast Asia, this assay is expected to facilitate the further development of a specific diagnostic tool for JEV infection.

## 1. Introduction

Japanese encephalitis virus (JEV) is a mosquito-borne orthoflavivirus that causes severe zoonotic diseases with significant public health and veterinary impacts across Asia and the Pacific region [1]. JEV is primarily transmitted by Culex mosquitoes and maintains an enzootic cycle involving wading ardeid birds as reservoir hosts and pigs as amplifying hosts, whereas humans are considered incidental dead-end hosts [2]. An estimated 3 billion people reside in JE-endemic areas, with approximately 68,000 clinical cases and 13,600–20,400 deaths reported annually worldwide [3,4]. However, the true disease burden is likely to be underestimated because of inadequate surveillance systems in many affected regions. JEV infection in swine is of particular concern, as pigs serve as the primary amplifying hosts, showing high viremia levels without apparent clinical signs, and thereby playing a critical role in viral transmission to humans. JEV infection causes abortion, stillbirth, and mummified fetuses in pregnant sows [5] and orchitis in boars [6], leading to huge economic losses in the pig industry [7]. It has been reported that JEV can also infect sheep and be isolated from sheep brains [8]. This new finding indicates an expansion of the host spectrum, which presents new risks and challenges for the prevention and control of JEV. Therefore, efficient surveillance and early detection of JEV infection in both humans and animals are essential for timely outbreak response and the implementation of control measures.

JEV belongs to the genus *Orthoflavivirus* of the family *Flaviviridae*. The JEV genome encodes three structural proteins (C, prM, and E) and seven nonstructural (NS) proteins (NS1, NS2A, NS2B, NS3, NS4A, NS4B, and NS5). Among these, the NS1 glycoprotein is a highly conserved, multifunctional nonstructural protein with a molecular mass of 46–55 kDa, depending on its glycosylation status [9,10]. NS1 exists in multiple oligomeric forms: an intracellular dimer associated with the viral replication complex, a cell-surface-associated form, and a secreted hexameric lipoparticle that circulates in the blood [10,11]. This protein plays an essential role in viral RNA replication, immune evasion, and pathogenesis. NS1 is abundantly secreted into the serum of infected individuals during the acute phase of infection, making it an ideal early diagnostic biomarker for orthoflavivirus infections [12]. The detection of NS1 antigen has been widely and successfully applied for the early diagnosis of dengue virus (DENV) infection, with commercial NS1-based ELISA kits demonstrating high sensitivity and specificity in acute-phase serum samples [13]. Given the structural and functional similarities among orthoflaviviruses, NS1-based diagnostic strategies have also been explored for other members of this family, including the West Nile virus (WNV) and Zika virus (ZIKV) [14,15].

Several studies have reported the development of NS1-based immunoassays for JEV detection. Kumar et al. developed a monoclonal antibody-based NS1 antigen capture ELISA using a rabbit polyclonal capture antibody and a mouse monoclonal detector antibody, which showed 97% concordance with real-time RT-PCR when tested with 120 acute-phase serum and 80 cerebrospinal fluid (CSF) samples from human patients [16]. Li et al. described another NS1 antigen capture assay using a panel of monoclonal antibodies, demonstrating the ability to detect NS1 protein in human serum samples at concentrations as low as 2 ng/mL, with sensitivity across multiple JEV genotypes [17]. While these studies demonstrated the feasibility of NS1-based JEV diagnosis, the assays were largely focused on either human clinical samples or required the use of polyclonal antibodies as capture or detection reagents. Furthermore, reports on the application of a single monoclonal antibody (mAb) serving as both the capture and detection antibody for JEV NS1 antigen capture ELISA in animal infection detection are scarce. In swine, which are key sentinels for predicting JEV outbreaks in humans, the development of simple, rapid, and cost-effective diagnostic methods is urgently needed for large-scale serosurveillance and infection monitoring.

In this study, we employed a mammalian cell line expression system to produce the JEV NS1 protein and generated a novel mAb (20B6) that specifically recognizes the JEV NS1 protein. Using this single mAb as both the capture and detection antibodies, we established a double-antibody sandwich antigen capture ELISA method for the detection of JEV NS1 protein. The performance of this assay was evaluated using clinical samples from experimentally infected and field-collected animals. Our findings suggest that this newly developed method provides a convenient, specific, and sensitive tool for the detection of NS1 protein in JEV-infected animals, with potential applications in both veterinary surveillance and epidemiological monitoring of this important zoonotic pathogen.

## 2. Materials and Methods

### 2.1. Cells, Viruses and Serum

Baby hamster kidney cell line BHK-21 cells (ATCC, CCL-10) and porcine kidney cell line PK15 cells (ATCC, CCL-33) were cultured in Dulbecco’s modified Eagle’s medium (DMEM; Gibco, Beijing, China). The mouse myeloma cell line SP2/0 (ATCC, CRL-1581) was maintained in RPMI 1640 medium (Gibco, Beijing, China). All cell culture media were supplemented with 10% FBS (Cellmax, Beijing, China). JEV (HLJ strain) and ZIKV [18] were propagated and titrated in BHK-21 cells. Viruses of ASFV, PRV, EMCV, CSFV, PRRSV, PEDV, TGEV, PDCoV, SADS-CoV, and SVA were donated by different research teams at the Harbin Veterinary Research Institute. The DENV 1–4 samples used in this study were generously gifted by Professor Cheng-feng Qin. Serum samples from pigs were obtained from the Diagnostic Center of Harbin Veterinary Research Institute. Specific pathogen-free (SPF) pig serum was used as a negative control.

### 2.2. Sequence Alignment of NS1 Proteins of Different Orthoflaviviruses

The sequences of NS1 protein of orthoflaviviruses were obtained from public databases (GenBank and UniProt) based on accession numbers: JEV P3 (U47032.1), JEV SA14 (P27395.1), JEV SA14-14-2 (MK585066), WNV MS1 (Q9Q6P4), KUNV (MF289571.1), USUV (AWC68492.1), MVEV (MN933859), SLEV (P09732.2), DENV 1 (P17763.2), DENV 2 (P29990.1), DENV 3 (YP_001621843.1), DENV 4 (P09866.2), ZIKV MR766 (AY632535), ZIKV Natal RGN (KU527068.1), Yellow fever virus (YFV) 17D (NP_041726.1), Wesselsbron virus (ABI54474.1), Tick-borne encephalitis virus (TBEV) (NP_043135.1), and Powassan virus LB (ACD88752.1). These sequences were aligned using the MUSCLE method, and a phylogenetic tree was constructed using the neighbor-joining method.

### 2.3. Protein Expression and Purification

Codon-optimized cDNA encoding the NS1 proteins of JEV, WNV, KUNV, USUV, MVEV, and SLEV were synthesized and cloned into the expression vector pCAG-neo, as previously described [19]. The recombinant pCAG-JEV-NS1-His plasmids were linearized with the restriction enzyme Ssp I (Takara, Beijing, China) and transfected into BHK-21 cells using the ExFect Transfection Reagent (Vazyme, Nanjing, China). Forty-eight hours after transfection, the cells were cloned into 96-well plates using the limited dilution method and cultured in G418-containing medium. The supernatants of cell lines were analyzed by Western blotting (WB) with a mouse anti-6×His tag monoclonal antibody (Proteintech, Wuhan, China) to select those exhibiting high levels of NS1 protein expression. The selected high-expression cell line was further confirmed as monoclonal by IFA, and its supernatant was used to purify recombinant NS1 proteins with Ni Sepharose excel (Cytiva, Uppsala, Sweden). The recombinant NS1 protein was detected for glycosylation following the manufacturer’s instructions provided with the Protein Deglycosylation Kit (NEB, Ipswich, MA, USA). The oligomeric form of the purified NS1 protein was analyzed by Western blotting after heating or treatment with β-mercaptoethanol.

### 2.4. SDS-PAGE and Western Blot

Purified protein samples, supernatants of cell culture medium, or lysates of infected cells were separated on 12% SDS-PAGE using the One-Step PAGE Gel Fast Preparation Kit (Vazyme, Nanjing, China) in a protein electrophoresis apparatus (Bio-Rad, Hercules, CA, USA) with a procedure running at 80 volts for 20 min and then at 140 volts for 1 h. The gels containing proteins were stained with Coomassie Brilliant Blue (Biodragon, Suzhou, China) or subsequently transferred onto nitrocellulose membranes (PALL, Pensacola, FL, USA) using a semi-dry transfer apparatus (Bio-Rad, Hercules, CA, USA) with a procedure running at 15 volts for 1 h. The membranes were blocked for two hours in 5% skimmed milk and then incubated with JEV NS1 specific monoclonal antibodies [20] or an anti-6×His monoclonal antibody (Proteintech, Wuhan, China) at 37 °C for 1 h. After washing three times with PBST for 5 min each, the membranes were incubated with IRDye^®^ 800CW donkey anti-mouse IgG (LI-COR, Lincoln, NE, USA) at room temperature for 1 h. After washing three times, the protein bands were detected using an Odyssey CLX Infrared Imaging System (LI-COR Biosciences, Lincoln, NE, USA).

### 2.5. Enzyme-Linked Immunosorbent Assay (ELISA)

The 96-well microplates (Corning, Kennebunk, ME, USA) were coated with purified NS1 protein or monoclonal antibodies diluted with carbonate buffer (0.05 M, pH 9.6) and incubated at 4 °C overnight. After washing three times with PBST for 3 min each, the microplates were blocked at 4 °C overnight or at 37 °C for 2 h. Sera from mice or pigs, monoclonal antibodies, supernatants of transfected cells, supernatants and lysates of virus-infected cells, or tissue lysates of JEV-infected mice were added to microplates and incubated at 37 °C for 2 h. The plates were subsequently incubated with HRP-conjugated goat anti-mouse IgG (H + L) (Invitrogen, Frederick, MD, USA) secondary antibody or HRP-labeled monoclonal antibody 20B6 at 37 °C for 1 h. After washing, 3,3′,5,5′-Tetramethylbenzidine (Beyotime, Shanghai, China) was added to the plates for substrate reaction, and 2 mol/L H_2_SO_4_ was used to stop the reaction. The optical density at 450 nm was measured using an ELISA plate reader (Bio-Rad, Hercules, CA, USA).

### 2.6. Immunofluorescence Assay

The cells expressing NS1 protein or JEV-infected cells were fixed with 4% paraformaldehyde at room temperature (RT) for 30 min. After washing twice with phosphate-buffered saline (PBS), the fixed cells were permeabilized with 0.1% Triton X-100 (Sigma-Aldrich, St. Louis, MO, USA) for 10 min. After blocking with 4% bovine serum albumin (BSA) at 37 °C for 30 min, the cells were incubated with the monoclonal antibody at 37 °C for 1 h. After washing with PBS, the cells were incubated with DyLight 488 Goat Anti-Mouse IgG (Abbkine, Wuhan, China) at RT for 1 h. The cells were observed using an Inverted Fluorescence Microscope (Life, EVOS FL, Carlsbad, CA, USA).

### 2.7. Stability Analysis of the JEV NS1 Dimer

The medium harvested from JEV-NS1 expressing cells or JEV-infected cells was clarified by centrifugation at 13,680× *g* for 5 min. The clarified supernatant was aliquoted into microcentrifuge tubes. To assess the thermostability of the NS1 dimer, aliquots were incubated at various temperatures for 30 min. The temperatures tested were 25 °C (approximately room temperature), 37 °C, 40 °C, 50 °C, 56 °C, 60 °C, 70 °C, 80 °C, 85 °C, 90 °C, 95 °C, and 100 °C. The treated samples were then mixed with the electrophoresis loading buffer (without any further boiling treatment) and promptly analyzed by Western blotting. To evaluate the sensitivity of the NS1 dimer to pH, aliquots of cell supernatants (original pH 7.4) were adjusted to various pH values. The target pH values were 2, 3, 4, 5, 6, 7.4 (untreated control), 9, 10, 12, and 13. The pH of each aliquot was monitored in real time and precisely adjusted using a Mettler Toledo MP220 pH meter. Once the desired pH was reached, the samples were incubated at room temperature for 30 min. After the above treatments, samples were promptly analyzed using Western blotting.

### 2.8. Generation of Monoclonal Antibodies Against the JEV NS1 Protein

Female BALB/c mice were initially immunized with purified NS1 protein emulsified with Freund’s complete adjuvant (Sigma-Aldrich, St. Louis, MO, USA) via subcutaneous injection. Three weeks after the first immunization, a booster immunization was performed with the NS1 protein emulsified in Freund’s incomplete adjuvant (Sigma-Aldrich, St. Louis, MO, USA). Three weeks later, a third dose was administered using the same dosage and method as for the second dose. Three days before cell fusion, the mice were treated with purified NS1 protein without adjuvant by intraperitoneal injection. Spleen cells from treated mice were fused with the mouse myeloma cell line SP2/0 with PEG to generate hybridoma cells, according to a previously described protocol [21]. BALB/c mice, first primed with mineral oil (Sigma, St. Louis, MO, USA), were inoculated with hybridoma cells via intraperitoneal injection to produce ascites. Monoclonal antibodies were purified from the collected ascites using PurKine™ Protein A/G Resin 4FF (Abbkine, Wuhan, China). Antibody subtypes were determined using a Mouse Antibody Typing Kit (containing κ and λ subtypes; Thermo Fisher Scientific, Rockford, IL, USA). Protein A/G magnetic beads (MCE, Monmouth Junction, NJ, USA) were bound to monoclonal antibodies to compare their ability to capture the NS1 protein from the supernatant of infected JEV cells.

### 2.9. Development and Optimization of the Capture ELISA for JEV NS1 Protein

Capture ELISA was developed by selecting appropriate combinations of capture and detection antibodies. The optimal coating concentration of the capture antibody and dilution of the enzyme-labeled detection antibody were determined by checkerboard titration. A series of optimization experiments were performed to select the appropriate coating, blocking, and dilution buffers, as well as to determine the optimal incubation temperature and time for each step. Subsequently, the incubation time and temperature were optimized for the antigen and enzyme-labeled antibody, respectively. A panel of 83 specific pathogen free (SPF) pig serum samples were tested to establish the cut-off value for the capture ELISA. The cut-off was calculated as the mean optical density (OD450) plus three standard deviations (mean + 3SD). Samples with OD450 values exceeding this threshold were considered positive. The assay was validated by assessing its specificity, limit of detection (LOD), and linear range. The reproducibility of the assay was evaluated by assessing the intra-assay and inter-assay precision. For intra-assay testing, five positive and two negative samples were tested in triplicate within a single run. For inter-assay reproducibility, the same samples were tested in three independent runs. BHK-21 cells were infected with Japanese encephalitis virus at a multiplicity of infection (MOI) of 0.1. The supernatants and cell lysates were collected to test for the NS1 protein of the Japanese encephalitis virus using capture ELISA.

### 2.10. JEV Infection Experiment in Mice

A total of 35 female BALB/c mice were randomly divided into an infection group (*n* = 29) and a non-infection control group (*n* = 6). Each mouse in the infection group was intraperitoneally inoculated with 10^5^ PFU of JEV and intracranially inoculated with 0.03 mL of PBS, while each mouse in the control group was intraperitoneally inoculated with 0.2 mL of PBS and intracranially inoculated with 0.03 mL of PBS. At each designated time point (days 0, 4, 5, 6, and 7 post-challenge), tissues of the heart, liver, spleen, lungs, kidneys, brain, and serum were collected from the mice. For each sampling time point, at least three mice were blindly selected for sample collection. The samples (0.1 g) were ground into a homogenate with 1 mL of PBS, and the homogenate was centrifuged at 10,000× *g* for 10 min. Supernatants were collected and used for ELISA and qPCR analyses. All data are presented as the mean ± standard deviation (SD) from at least three independent experiments. Statistical analyses were performed using GraphPad Prism software version 9.5.0.

### 2.11. Quantitative Real-Time Polymerase Chain Reaction (qRT-PCR)

Total RNA was extracted from the samples using TRIzol reagent (Invitrogen, Carlsbad, CA, USA) according to the manufacturer’s instructions. The RNA samples were reverse transcribed into cDNA using a Reverse Transcriptase M-MLV kit (Takara, Beijing, China) or stored at −80 °C. cDNA samples were tested using a quantitative real-time PCR kit (Takara, Beijing, China). As previously described [22], the reaction was performed on a Fluorescent Quantitative PCR Instrument (Applied Biosystems, QuantStudio 5) in a total volume of 20 μL, containing 10 μL of 2× Premix Ex Taq, 0.5 μM each of forward and reverse primers, 1 μL of cDNA template, and 7 μL of nuclease-free water. The thermal cycling conditions were as follows: initial denaturation at 95 °C for 5 min, followed by 40 cycles of denaturation at 95 °C for 10 s and annealing/extension at 60 °C for 40 s. All samples were analyzed in triplicate for technical reproducibility.

## 3. Results

### 3.1. Protein Expression and Purification

**Figure 1:**
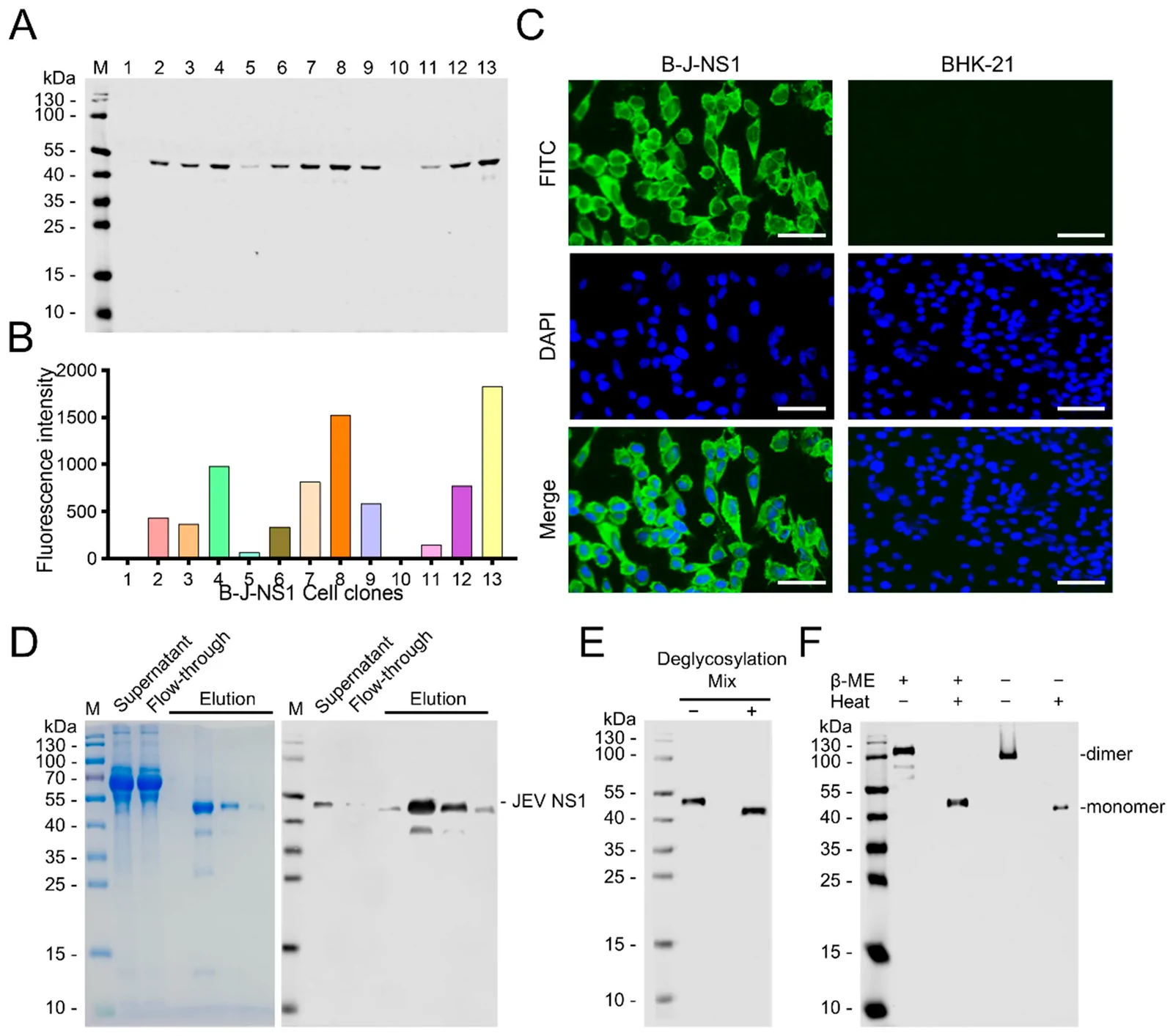
Expression and purification of JEV NS1 protein. BHK-21 cells were transfected with recombinant plasmids. After cloning and selection with G418, the supernatants of cloned cells were subjected to Western blotting with a JEV NS1 specific antibody (**A**), and the signals of the NS1 band were quantified using Image Studio software 3.1 (**B**). Clone #13, designated as B-J-NS1, which showed the highest expression level, was further analyzed by IFA. Cells were stained for NS1 protein (green) and nuclei (blue) (**C**), and this clone was selected to express the NS1 protein. Scale bar: 50 μm. The recombinant NS1 protein was purified from the culture medium of B-J-NS1 cells and subjected to SDS-PAGE and Western blotting analysis (**D**). The purified NS1 protein was analyzed using a Deglycosylation Mix (**E**). The sensitivity of recombinant NS1 protein to heat and β-mercaptoethanol (β-ME) was also analyzed (**F**).

To obtain the NS1 protein with post-translational modifications that were more similar to the native viral protein, stable mammalian cell line expression technology was adopted to produce the NS1 protein. First, BHK-21 cells were transfected with linearized plasmid pCAG-JEV-NS1-His. After limited-dilution cloning and selection with medium containing G418, the cell lines stably expressing the JEV NS1 protein were analyzed using Western blotting (Figure 1A). Through a quantitative comparison of NS1 expression levels (Figure 1B), the cell strain exhibiting the highest NS1 expression level was selected and verified by IFA (Figure 1C). The selected NS1 expressing cell line was designated as B-J-NS1. The recombinant NS1 protein was affinity chromatography purified with Ni Sepharose resin from the supernatant of the B-J-NS1 culture medium. The purified recombinant protein was analyzed by SDS-PAGE and verified by Western blotting (Figure 1D). To verify post-translational modification of the recombinant NS1 protein, the purified NS1 protein was first examined using a deglycosylation reagent. As shown in Figure 1E, the molecular weight of NS1 changed from approximately 55 kDa to approximately 40 kDa after deglycosylation treatment. These results demonstrate that the recombinant NS1 protein was glycosylated. We also surveyed the sensitivity of the recombinant NS1 protein to reducing agents (β-mercaptoethanol) and heat (boiling treatment) using Western blotting. It was found that the NS1 protein remained in a dimer form when no heat treatment was applied, with or without a reducing reagent. In contrast, the recombinant NS1 protein presented as a monomer under heat treatment with or without reducing reagents (Figure 1F). These results demonstrate that the recombinant NS1 protein exists as a thermosensitive, glycosylated, non-intermolecular disulfide bond-dependent dimer.

### 3.2. Stability Analysis of the JEV NS1 Dimer

**Figure 2:**
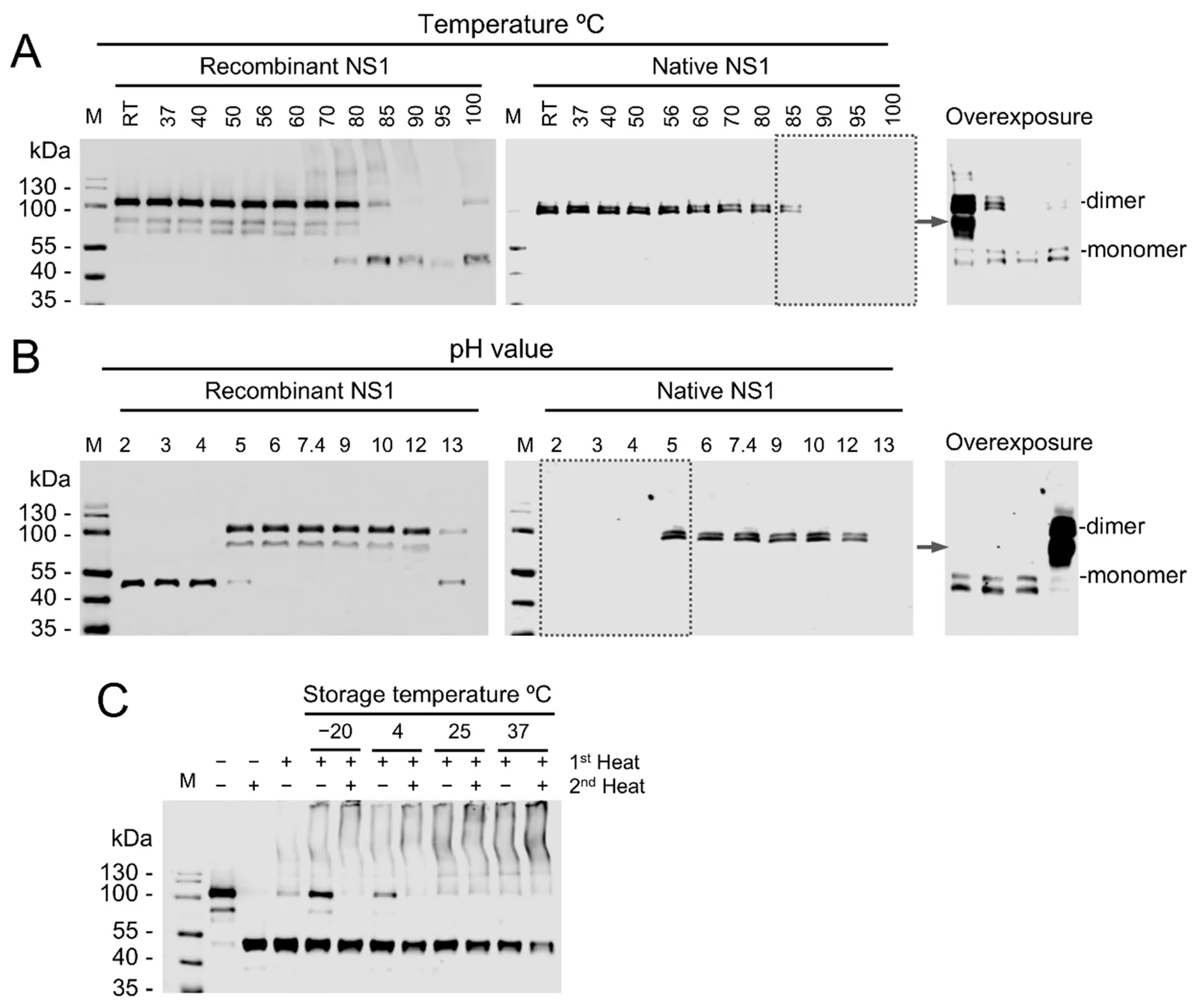
Analysis of the thermal and acid-base stabilities of recombinant and native JEV NS1 proteins. The recombinant NS1 protein and native secreted NS1 protein in the cell culture medium were treated at temperatures ranging from 25 °C to 100 °C. Samples treated at different temperatures were analyzed using Western blotting with a JEV NS1-specific antibody. The area within the dashed box is overexposed and displayed on the far-right panel (**A**). The recombinant NS1 protein and native secreted NS1 protein in the cell culture medium were treated at pH values ranging from 2 to 13. Samples without boiling treatment were immediately analyzed by Western blotting using a JEV NS1 specific antibody. The area within the dashed box was overexposed and is displayed in the far-right panel (**B**). Samples of recombinant NS1 protein with SDS-PAGE loading buffer with or without boiling treatment and heat-treated samples stored at different temperatures with or without heat treatment were analyzed by Western blotting (**C**).

To survey the stability of NS1 dimer under the common environmental temperature for sample collection, the recombinant NS1 protein and the native NS1 protein from infected cell culture medium were treated at temperatures ranging from 25 °C to 100 °C. Then NS1 was analyzed using Western blotting. As shown in Figure 2A, both recombinant NS1 protein and native NS1 proteins remained in dimer form below 80 °C. A small proportion of monomeric NS1 began to appear at 80 °C, and the dimeric form was almost completely dissociated at 90 °C (Figure 2A). These results demonstrate that the JEV NS1 protein exhibits considerable thermostability under common environmental temperatures for sample collection and storage. To investigate the influence of different pH values on the stability of NS1 dimers in the sample, recombinant NS1 protein and native NS1 proteins were treated with buffers with pH values ranging from 2 to 13. Western blotting results showed that both recombinant NS1 protein and native JEV NS1 remained in dimer form in the pH range of 5 to 12 (Figure 2B). These results indicate that NS1 protein is an appropriate target for detecting JEV infection. The NS1 protein can stably remain in the dimer form under common environmental temperatures and normal physiological pH values. Notably, during these experiments, it was observed that following heat denaturation, a portion of the monomeric NS1 was capable of spontaneously reassociating into dimers after storage, and this reconstruction process was temperature-dependent (Figure 2C).

### 3.3. Generation of JEV NS1 Specific mAbs

**Figure 3:**
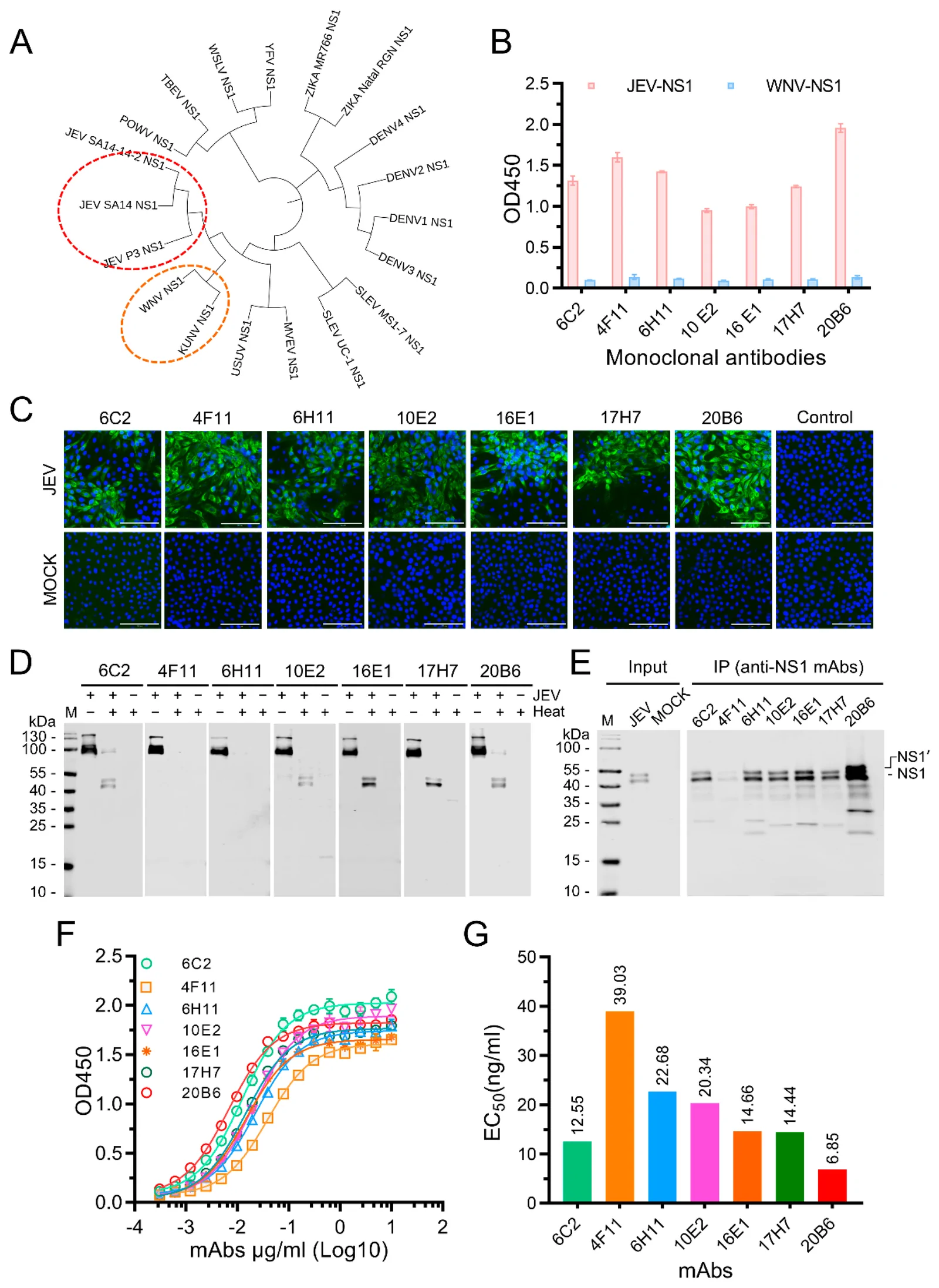
Generation and characterization of JEV NS1 specific monoclonal antibodies. (**A**) Amino acid sequence alignment analysis of orthoflavivirus NS1 protein. JEV NS1 is indicated by red dashed ellipses, while WNV NS1 and KUNV NS1, which are closely related, are indicated by orange dashed ellipses. After immunization and cell fusion, seven hybridoma cell clones were selected that could secrete JEV NS1 specific antibodies and did not react with WNV NS1 (**B**). The reactivity of the mAbs with JEV-infected PK15 cells was analyzed by IFA and cells were stained for NS1 protein (green) and nuclei (blue) (**C**) and Western blotting (**D**). The binding capacities of the mAbs to the native JEV NS1 protein in infected cells were evaluated by immunoprecipitation (**E**). The titers of purified mAbs were determined using ELISA (**F**), and the EC50 values were calculated (**G**).

To facilitate the screening of JEV NS1 specific mAbs, the amino acid sequences of the NS1 protein of relative orthoflavivirus were compared. Sequence alignment was performed, and a phylogenetic tree was constructed using the neighbor-joining method (Figure 3A). Sequence alignment revealed that the JEV NS1 protein shares a much higher sequence homology with the WNV NS1 protein. Therefore, the WNV NS1 protein was selected and used as a control antigen in hybridoma screening by ELISA. After cell fusion and ELISA screening, seven hybridomas that secreted mAbs specific to JEV NS1 were obtained and designated as 6C2, 4F11, 6H11, 10E10, 16E1, 17H17, and 20B6 (Figure 3B). The subtype of mAbs was identified using the Mouse Antibody Typing Kit, and the results revealed that six mAbs (6C2, 4F11, 6H11, 10E10, 16E1, and 17H17) belonged to the IgG1/κ subclass, whereas 20B6 was of the IgG2b/κ subclass. Monoclonal antibodies were purified from the supernatants of hybridomas cultured in serum-free medium using an affinity chromatography column. IFA results demonstrated that all seven mAbs recognized the native NS1 protein in JEV-infected PK15 cells, whereas no fluorescence was observed in uninfected cells (Figure 3C). Western blot analysis revealed that the mAbs recognized NS1 protein. mAbs 4F11 and 6H11 recognized only the oligomeric form of NS1, suggesting that they target epitopes formed across molecular chains. The other five mAbs (6C2, 10E10, 16E1, 17H17, and 20B6) recognized both monomeric and oligomeric NS1 (Figure 3D). A preliminary comparison of the NS1-binding capacities of the different mAbs was performed using immunoprecipitation. The results showed that mAb 20B6 displayed the highest level of NS1 capture in immunoprecipitation assays (Figure 3E). Furthermore, the titers of monoclonal antibodies were determined using ELISA (Figure 3F), and the EC_50_ values (median effect concentration) of the mAbs were calculated (Figure 3G). The results showed that 20B6 exhibited an EC_50_ value of 6.845 ng/mL, which was superior to those of the other mAbs.

### 3.4. Development and Optimization of JEV NS1 Protein Capture ELISA

**Figure 4:**
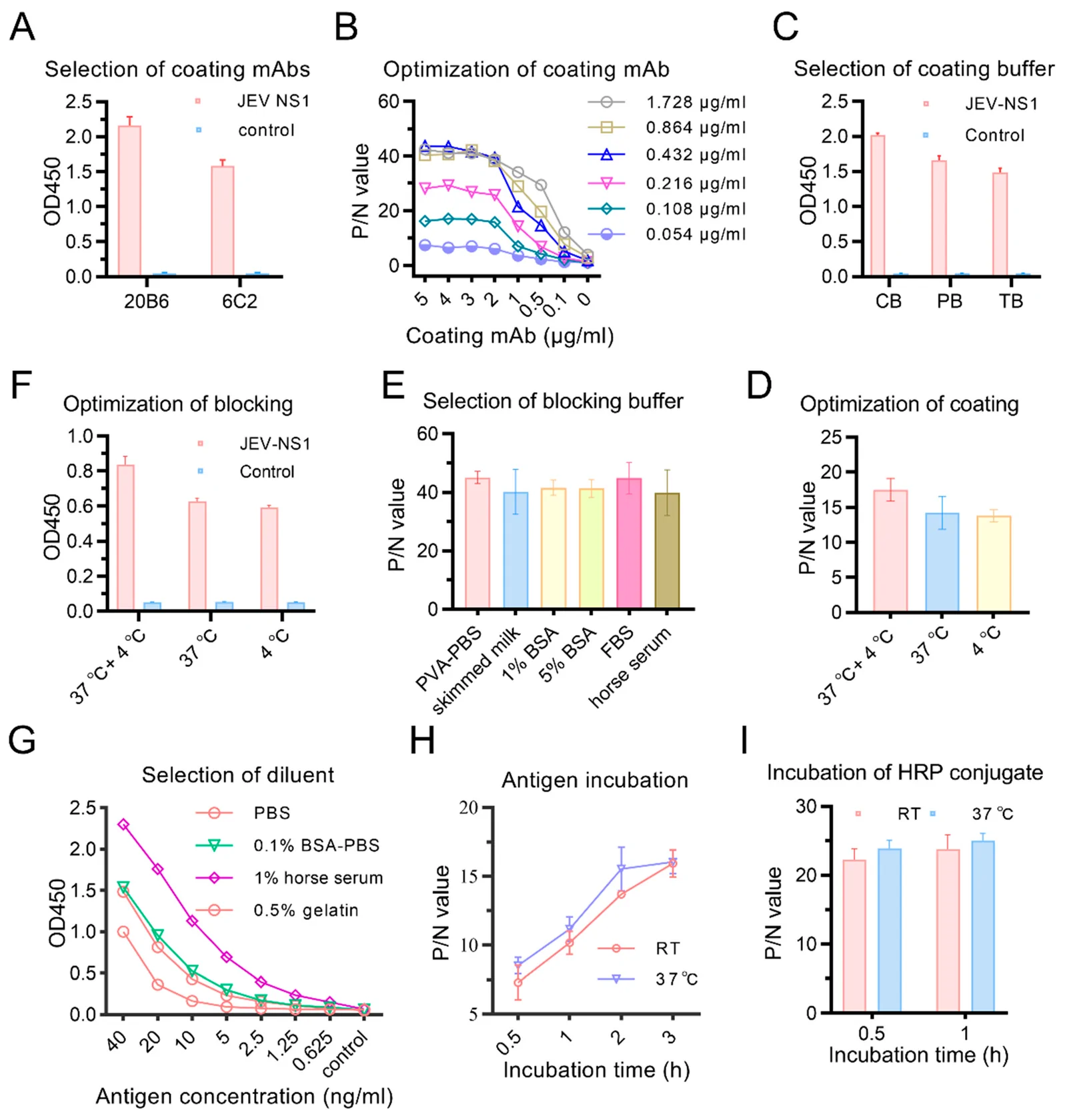
Development and optimization of JEV NS1 capture ELISA. To select the capture antibody, mAbs 20B6 and 6C2 were used as coating antibodies in the JEV NS1 capture ELISA (**A**). To determine the concentrations of coating and detecting antibodies, serially diluted mAb 20B6 was coated, and serially diluted HRP-conjugated mAb 20B6 was used as a detecting antibody in a capture ELISA (**B**). After determining the coating and detection antibodies, three coating buffers: carbonate buffer (CB), phosphate buffer (PB), and Tris-HCl buffer (TB) were compared in the NS1 capture ELISA (**C**). The coating process was further optimized for JEV NS1 capture ELISA (**D**). Six blocking buffers (0.5% PVA-PBS, 5% skimmed milk, 1% BSA, 5% BSA, 10% FBS, and 1% house serum) were compared for NS1 capture ELISA (**E**). The blocking process was also optimized (**F**). Sample diluents for the NS1 capture ELISA were also compared (**G**). Finally, the sample incubation time and temperature (**H**) and HRP conjugate incubation time and temperature (**I**) were optimized for the JEV NS1 capture ELISA.

In view of its superior binding capacity for NS1 compared to the other mAbs, 20B6 was adopted as the detection antibody and was conjugated with HRP (horseradish peroxidase) for the development of a capture ELISA. Meanwhile, 20B6 was found to be superior to the other antibodies as a capture antibody (Figure 4A). To achieve optimal assay performance, various parameters were systematically optimized. The optimal coating concentration of the capture antibody and dilution of the enzyme-labeled antibody were determined using checkerboard titration. When the 20B6 coating concentration reached 3 μg/mL, the curves for the detection antibody at all concentrations plateaued, and further increases in the coating concentration did not significantly enhance the signal. Additionally, when the detection antibody concentration reached 0.432 μg/mL, the response curve reached an optimal level, and further increases in the concentration of the detection antibody did not significantly enhance the signal (Figure 4B). Therefore, the optimal coating concentration of 20B6 was 3 μg/mL, and the optimal concentration of the enzyme-labeled antibody was 0.432 μg/mL. Furthermore, through a series of optimization experiments, the optimal coating, blocking, and dilution buffers were identified as carbonate buffer (CB; pH9.6), 0.5% PVA-PBS, and 1% horse serum, respectively (Figure 4C,E,G). The optimal coating process was determined to be incubation at 37 °C for 2 h, followed by incubation at 4 °C overnight. The optimal blocking process was 37 °C for 2 h (Figure 4D,F). The optimal antigen incubation condition was 37 °C for 2 h, and the optimal enzyme-labeled antibody incubation condition was 37 °C for 1 h (Figure 4H,I). The cut-off value was established by testing 83 SPF pig serum samples.

### 3.5. Specificity and Sensitivity of JEV NS1 Capture ELISA

**Figure 5:**
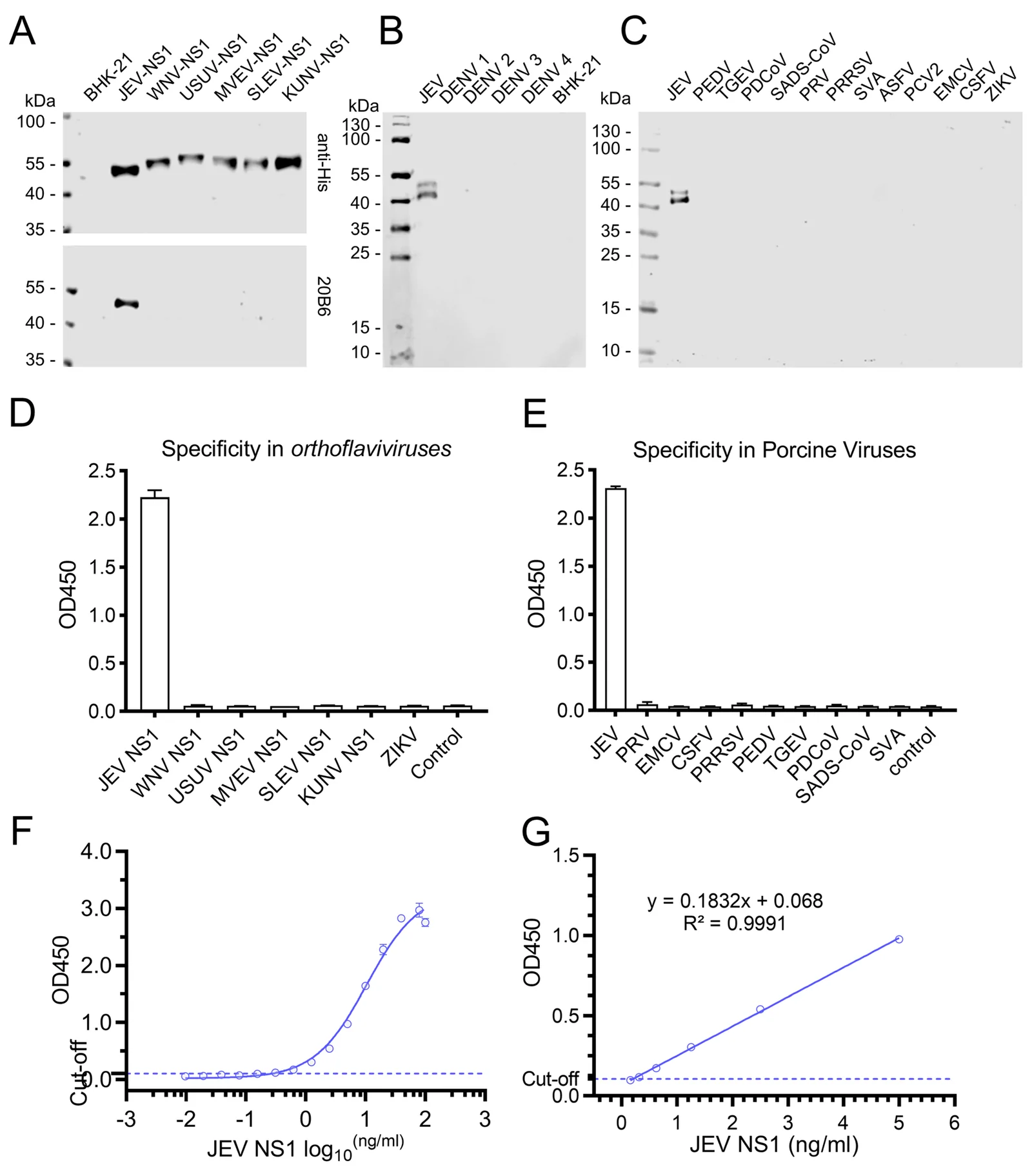
Specificity and sensitivity of JEV NS1 capture ELISA. The related orthoflavivirus NS1 proteins were expressed by plasmid transfection, and protein expression was verified by Western blotting with an anti-His tag antibody ((**A**), upper). The cross-reactivity of mAb 20B6 with the NS1 protein of related orthoflavivirus (WNV, KUNV, USUV, MVEV, SLEV, and native ZIKV NS1 proteins from infected cells in the (**C**) panel) was evaluated using Western blotting ((**A**), lower). The cross-reactivity of mAb 20B6 with DENV 1–4 was also evaluated by Western blotting (**B**). The cross-reactivity of mAb 20B6 with some common swine viral pathogens was also evaluated using Western blotting (**C**). The specificity of the JEV NS1 capture ELISA was evaluated using a homologous orthoflavivirus NS1 protein (**D**) and some common swine viral pathogens (**E**). The sensitivity of the capture ELISA was assessed by testing serial dilutions of the purified JEV NS1 protein (**F**). The linear correlation between the OD450 values and NS1 protein concentration range was evaluated (**G**).

To further verify the specificity of mAb 20B6, five more relative orthoflavivirus NS1 proteins were transiently expressed by plasmid transfection. Protein expression was verified by Western blotting using an anti-His tag antibody (Figure 5A upper panel). The Western blotting results showed that mAb 20B6 did not cross-react with the NS1 proteins of these relative orthoflavivirus (Figure 5A lower panel and Figure 5B). The mAb 20B6 also did not cross-react with 11 porcine viral pathogens and ZIKV (Figure 5C). The specificity of the established capture ELISA was evaluated. As shown in Figure 5D, no cross-reactivity was observed with the NS1 protein from other orthoflaviviruses, including WNV, KUNV, USUV, MVEV, SLEV, and ZIKV. Furthermore, to assess potential interference in clinically relevant scenarios, the assay was tested against a panel of common porcine pathogens, including PEDV, TGEV, PDCoV, SADS-CoV, PRV, PRRSV, SVA, ASFV, PCV2, EMCV, and CSFV. The results demonstrated that the assay exhibited excellent specificity, with no non-specific reactivity detected against any of the viruses (Figure 5E). To determine the sensitivity of the established capture ELISA, a series of diluted JEV NS1 proteins were tested. The assay exhibited a sensitivity of 0.313 ng/mL, and the linear range was 0.313–5 ng/mL with a linear relationship (R^2^ = 0.99) (Figure 5F,G). The reproducibility of the assay was evaluated by assessing intra-assay and inter-assay precision. For intra-assay, the range of the coefficient of variation (CV) was 0.678–4.831%. For inter-assay reproducibility, the range of CV was determined to be 0.549–3.266%. All the coefficients of variation were less than 10%.

### 3.6. Detection of NS1 Protein in JEV Infected BHK Cells

**Figure 6:**
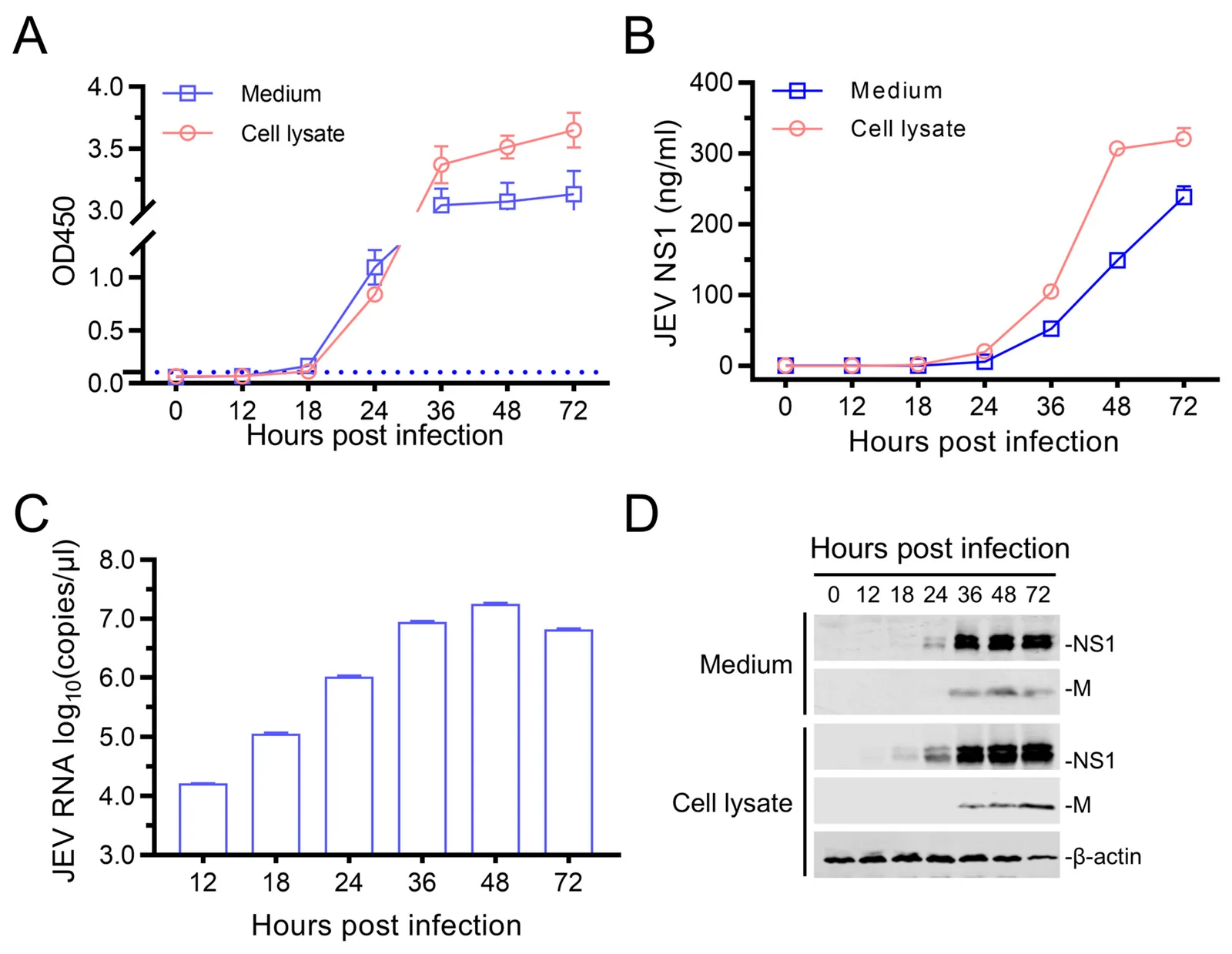
NS1 expression dynamics in JEV-infected BHK-21 cells. BHK-21 cells were infected with JEV at a multiplicity of infection (MOI) of 0.01. At the designated time points, NS1 protein in the cell culture medium and cell lysates was detected using NS1 capture ELISA (**A**). Furthermore, NS1 protein levels in the cell culture medium and cell lysate were quantitatively calculated (**B**). Correspondingly, the viral RNA levels in the JEV-infected cell culture medium were tested using quantitative RT-PCR (**C**). NS1 and M proteins in JEV-infected cell medium and cell lysate were detected by Western blotting (**D**).

Capture ELISA was used to test the kinetics of NS1 expression and secretion in BHK-21 cells infected with JEV. As shown in Figure 6A, the results indicated that the NS1 protein could be detected as early as 18 h after infection in both cell lysates and cell culture medium. NS1 protein levels were quantified according to an established standard curve (Figure 6B). The JEV NS1 protein accumulated steadily in the culture medium, reaching the highest level of approximately 250 ng/mL during the observation period. The released viral RNA in the cell culture medium was detected using qRT-PCR. Viral RNA was detected at the first sample collection time point, 12 h post-infection (Figure 6C). The viral proteins NS1 and M in the culture medium and cell lysate were monitored using Western blotting. The results showed that the NS1 protein could be detected earlier than the M protein in both the medium and cell lysate (Figure 6D).

### 3.7. Detection of NS1 Protein in JEV Infected Mice

**Figure 7:**
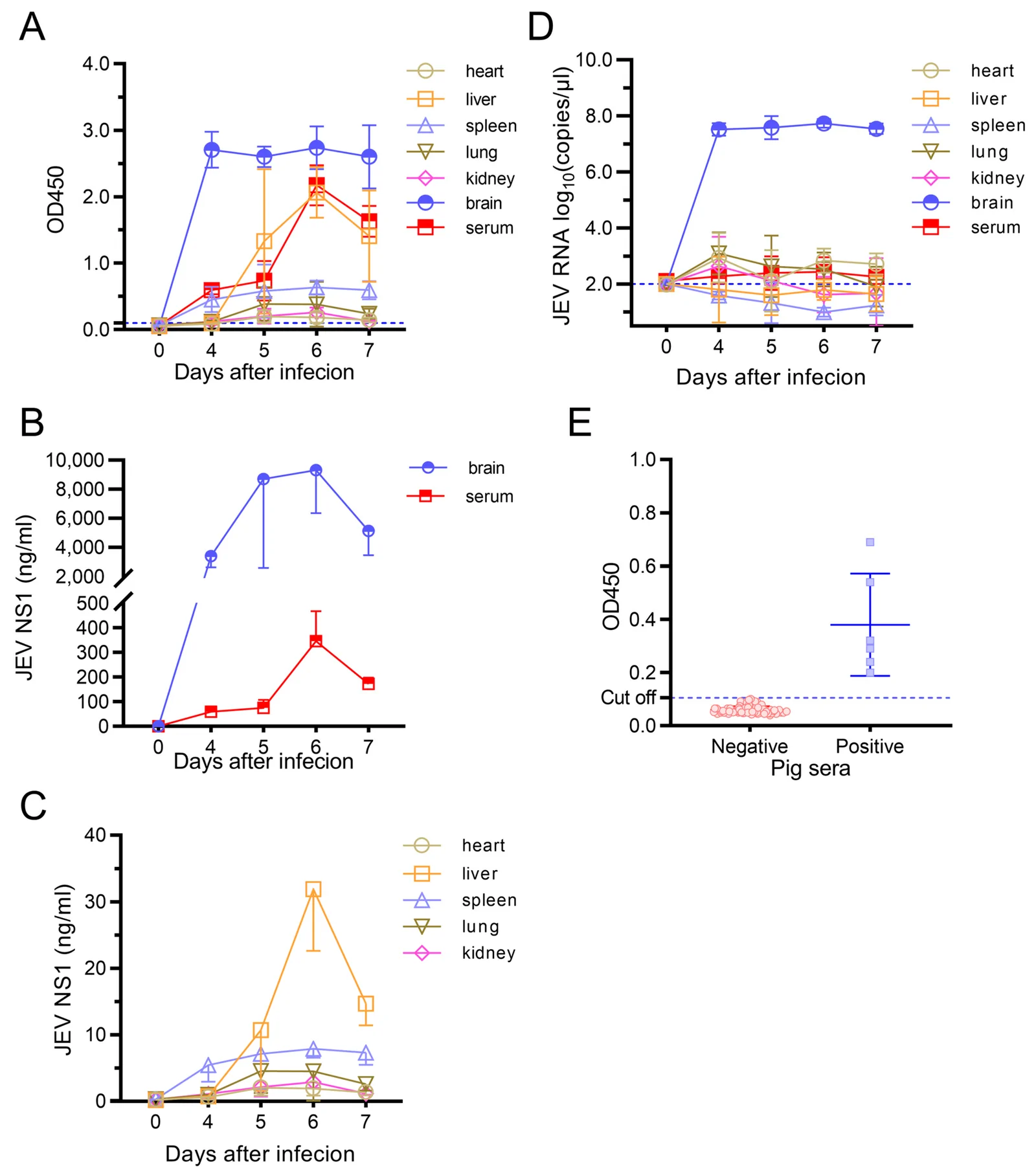
Detection of NS1 protein in the tissues and sera of JEV-infected animals. (**A**) Detection of NS1 protein in the serum and tissues of JEV-infected mice using capture ELISA. NS1 protein levels in the serum and tissues were quantitatively calculated (**B**,**C**). (**D**) Quantitative RT-PCR analysis of viral RNA in the serum and tissues of JEV-infected mice. (**E**) Detection of NS1 protein in porcine serum samples using the established capture ELISA.

To evaluate the practicability of this method and select appropriate tissue samples, the hearts, livers, spleens, lungs, kidneys, brain tissues, and sera of JEV-infected mice were collected. On the 4th day after infection, the JEV NS1 protein was detected in the brain and serum. From the 5th to 7th day post-infection, NS1 levels increased in all tissue samples, with the brain, serum, and liver exhibiting a significant increase (Figure 7A). The highest NS1 protein content was found in brain tissue, followed by serum. The peak mean concentrations of NS1 protein in the brain, serum, and liver were 9.32 μg/mL, 346.25 ng/mL, and 31.89 ng/mL, respectively (Figure 7B,C). NS1 levels in other tissues were relatively low (Figure 7C). The viral load in the brain was markedly higher than that in other tissues, consistent with the neurotropic nature of JEV (Figure 7D).

### 3.8. Detection of JEV Infection with Pig Sera by NS1 Capture ELISA

A total of 116 swine serum samples were tested using capture ELISA. Data are presented as the mean of three independent biological replicates. Six samples were ELISA-reactive and 110 were non-reactive, yielding an ELISA-reactive rate of 5.17% (Figure 7E).

## 4. Discussion

The diagnosis of orthoflavivirus infections primarily involves etiological and serological methods. Among the etiological approaches, virus isolation, quantitative PCR (qPCR), and viral antigen detection are commonly employed. While virus isolation remains a reliable method for the definitive diagnosis of orthoflavivirus infection, its application is constrained by stringent technical requirements, prolonged culture periods, high costs, and suboptimal sensitivity. The qPCR method offers high sensitivity, but it requires sophisticated instrumentation and specialized laboratory facilities. The NS1 protein is the predominant target for orthoflavivirus antigen detection [10]. Critically, all these etiological methods face a common challenge: the transient nature of viremia during orthoflavivirus infection and low viral loads, meaning the pathogen persists in the patient’s bloodstream for only a brief period [23]. Common serological methods include virus neutralization tests (VNT), hemagglutination inhibition (HI) assays, and detection of IgM and IgG antibodies. These methods offer advantages such as high efficiency, time savings, good sensitivity, and suitability for large-scale tests. However, these methods have some drawbacks. There is a high degree of amino acid sequence homology among orthoflaviviruses, which leads to the production of antibodies with cross-reactivity during immune responses. The application of serological methods for orthoflavivirus detection is inevitably associated with cross-reactivity, compromising diagnostic accuracy [24,25]. In light of these challenges, the nonstructural protein 1 of orthoflavivirus offers a compelling alternative target with the potential to overcome these constraints. First, unlike structural proteins, its levels do not directly decline with viral load. Second, the specificity of the assay can be achieved through rigorous screening of antibodies specific to the target protein.

The NS1 protein of orthoflavivirus is initially synthesized as a soluble monomer in the cytoplasm, followed by post-translational modifications within the endoplasmic reticulum and Golgi network, which facilitates rapid dimerization. Subsequently, it is transported to the cell surface and secreted into the extracellular space [9]. The secreted form of NS1 (sNS1) is believed to be a lipid-associated hexamer that circulates at high levels in the blood of infected patients [12]. It is important to note that the formation and secretion of NS1 structure are highly dependent on the glycosylation status of NS1 [26]. Thus, we produced the recombinant NS1 protein in mammalian cells, which more closely resembled its native form compared to prokaryotic expression systems lacking post-translational modifications [27] or baculovirus-insect cell systems deficient in key enzymes required for long-chain glycan processing [28,29]. The hexameric and tetrameric forms of the NS1 protein are relatively unstable, whereas the dimeric form exhibits excellent stability, and the dimer of NS1 only dissociates into monomers under extreme conditions, such as high temperature or acidity [10,30]. Moreover, we demonstrated that the dimer of both recombinant NS1 protein and native JEV NS1 protein retained its structural integrity at temperatures up to 80 °C and within a pH range of 5 to 12. This observation indirectly validated the superiority of our mammalian expression platform and indicated that the NS1 protein in the samples remains at least in its dimeric form under natural sampling conditions and after routine processing, thereby ensuring the stability of the detection target. The structural characteristics of the NS1 protein provided a foundation for the development of a capture ELISA utilizing a single antibody as both the capture and detection reagent. This approach may simplify the method development process, reduce costs, and ensure consistent recognition of the JEV NS1 dimer without the need for paired antibody screening.

The *Orthoflavivirus* members exhibit a high degree of similarity in both amino acid sequences and structural conformations [31]. The NS1 protein of JEV shares approximately 74% amino acid homology with that of WNV. This substantial sequence conservation frequently causes cross-reactivity in conventional detection methods, consequently impairing diagnostic accuracy to a significant extent. For instance, a notable example occurred in Xinjiang, China, where a cluster of febrile and meningitis cases was initially diagnosed as JEV infection due to positive JEV IgM antibody test results. However, because of the poor specificity of the diagnostic assay, further rigorous investigation revealed that the outbreak was actually caused by WNV [32,33]. Similarly, a 2018 metagenomic sequencing study identified genetic fragments of both DENV and JEV in mosquito pools collected from Yunnan Province, suggesting the co-circulation of these two pathogens in this region [34]. Furthermore, a subsequent investigation into the distribution of JEV in Yunnan demonstrated that DENV outbreaks occurred in areas with prolonged JEV endemicity, as confirmed by NS1 antigen detection, IgM antibody assays, and real-time RT-PCR [35]. These findings underscore the diagnostic challenges posed by orthoflavivirus co-circulation. To address this issue, we established a specific ELISA based on a dimeric JEV NS1 protein. The method showed high specificity, with no cross-reactivity observed against NS1 proteins of other orthoflaviviruses that share high sequence homology with JEV NS1, including WNV, KUNV, USUV, MVEV, SLEV, and ZIKV. In addition, the mAb 20B6 used in this assay showed no cross-reactivity with dengue virus serotypes 1–4. This highlights the value of this method in regions where multiple orthoflaviviruses co-circulate.

To evaluate the practical applicability of this method, we detected NS1 protein in BHK cells infected with JEV. NS1 protein was detected as early as 18 h post-infection, and its abundance correlated positively with increasing viral titers. These findings demonstrate that NS1 is continuously present throughout the course of JEV infection, providing a robust theoretical foundation for the detection of JEV infection. To further simulate the clinical detection of JEV infection and determine the optimal sample type for testing, we employed a mouse infection model. The highest viral loads and NS1 protein levels were observed in the brain tissue, consistent with the neurotropic nature of JEV. Notably, although the viral titers in mouse serum were relatively low, NS1 protein levels were substantially higher. This finding suggests that NS1 protein detection in serum could be used as a complement to nucleic acid detection for diagnosing JEV infection. Collectively, this work would benefit the development of a specific diagnostic tool for Japanese encephalitis.

## 5. Conclusions

In summary, this study successfully purified JEV NS1 protein. The protein was used to immunize mice to generate and select a monoclonal antibody (20B6) with a strong binding affinity. Based on the mAb 20B6, we established a highly specific and sensitive capture ELISA for JEV NS1 during the laboratory development stage. This method holds promise for addressing the issue of orthoflavivirus cross-reactivity.

## Data Availability

All data generated or analyzed during this study are included in this article.
